# Bioactive Lipids in MSCs Biology: State of the Art and Role in Inflammation

**DOI:** 10.3390/ijms22031481

**Published:** 2021-02-02

**Authors:** Sara Casati, Chiara Giannasi, Stefania Niada, Roberta F. Bergamaschi, Marica Orioli, Anna T. Brini

**Affiliations:** 1 Dipartimento di Scienze Biomediche, Chirurgiche ed Odontoiatriche, Università degli Studi di Milano, 20133 Milan, Italy; chiara.giannasi@unimi.it (C.G.); roberta.bergamaschi@unimi.it (R.F.B.); marica.orioli@unimi.it (M.O.); anna.brini@unimi.it (A.T.B.); 2 IRCCS Istituto Ortopedico Galeazzi, 20161 Milan, Italy; stefania.niada@grupposandonato.it

**Keywords:** bioactive lipids, lipidomics, mesenchymal stem cells, inflammation, osteoarthritis

## Abstract

Lipidomics is a lipid-targeted metabolomics approach that aims to the comprehensive analysis of lipids in biological systems in order to highlight the specific functions of lipid species in health and disease. Lipids play pivotal roles as they are major structural components of the cellular membranes and energy storage molecules but also, as most recently shown, they act as functional and regulatory components of intra- and intercellular signaling. Herein, emphasis is given to the recently highlighted roles of specific bioactive lipids species, as polyunsaturated fatty acids (PUFA)-derived mediators (generally known as eicosanoids), endocannabinoids (eCBs), and lysophospholipids (LPLs), and their involvement in the mesenchymal stem cells (MSCs)-related inflammatory scenario. Indeed, MSCs are a heterogenous population of multipotent cells that have attracted much attention for their potential in regulating inflammation, immunomodulatory capabilities, and reparative roles. The lipidomics of the inflammatory disease osteoarthritis (OA) and the influence of MSCs-derived lipids have also been addressed.

## 1. Introduction: Lipidomics and Lipids Mediated Inflammation in Mesenchymal Stem Cells

### 1.1. Lipidomics

The lipidome is defined as the complete set of lipids present within a cell, a tissue, or an organism [1,2]. In the last decades, it has become clear that the lipidome, as well as the transcriptome and the proteome, is in a dynamic balance and it can be affected by physio-pathological conditions, stimuli, and changes in diet [3,4]. Lipidomics is a relatively new “-omics” that characterizes, identifies, and quantifies the lipidome and its metabolic pathways and other networks that are involved within different biological mechanisms [5]. With analytical approaches, such as thin-layer chromatography (TLC) and gas chromatography (GC), lipidomics was able to develop new diagnostic tools and therapeutic strategies [6]; but it was with the advent of the next-generation mass spectrometry (MS) that there have been significant advances in the field of lipidomics [7,8,9,10,11]. In a typical lipidomic workflow, lipids are extracted from the biological matrices using organic solvents and analyzed by direct infusion into a mass spectrometer (technique known as “shotgun” lipidomics), or separated by liquid (LC) or gas chromatography (GC), prior to detection by MS. These two approaches are complementary, since the “shotgun” method allows a larger lipid profiling by simultaneous identification of several classes of lipids, meanwhile LC or GC/MS enable a more targeted analysis with the detection of structurally similar lipids belonging to a single class [12,13,14]. In both methods, the quantification is performed using a ratio against internal standard(s), which is routinely added for sample normalization and matrix effect influence correction. Internal standard structures and physicochemical properties are representative of the endogenous lipid species of interest and are added at the earliest possible step during sample preparation. For shotgun lipidomics, a semi-quantification is generally possible by using exogenous lipids representative of the main lipid classes of interest; whereas for targeted lipidomics, labeled lipids (i.e., deuterated internal standards) should be included for absolute quantification. In the last few years, the aforementioned advanced analytical techniques have led to multiple improvements in lipidomics, particularly in the extraction methods and bioinformatics. These enhancements have allowed important goals, such as the identification of several lipid-based biomarkers, useful as diagnostic tools [5,10]. However, the number of lipidomics studies in the field of mesenchymal stem cells (MSCs) remains rather limited, especially when compared to the numerous investigations about their transcriptome and proteome. Thus, the objective of the current review was to focus on recently highlighted roles of specific bioactive lipid species and their involvement in the MSCs-related inflammatory scenario.

### 1.2. Involvement of MSC in Inflammatory Processes

MSCs are non-hematopoietic multipotent progenitor cells with the ability to differentiate into different mesodermal lineages including osteocytes, chondrocytes, and adipocytes [15,16]. The three criteria adopted by the International Society for Cellular Therapy to define and identify MSCs are: (1) MSCs must be adherent to plastic under standard culture conditions; (2) their phenotypes must present the expression of CD105, CD73, and CD90 and lack the expression of the hematopoietic cell surface markers CD45, CD34, CD14 or CD11b, CD79α, or CD19 and HLA-DR; (3) they must be able to differentiate under stimulation in vitro into osteoblasts, adipocytes, and chondroblasts [17,18]. MSCs are currently being studied in many preclinical and clinical applications. In particular, they have attracted the scientific interest for their ability to regulate inflammatory processes and promote tissue repair due to their multi-lineage differentiation potential, pro-angiogenic characteristics, and immune-modulatory properties [19,20,21]. Recently, MSC-based treatment has been proposed as a suitable therapeutic approach for the severe acute respiratory infection caused by the corona virus SARS-CoV-2. In the COVID-19 scenario, where the immune system produces large amounts of inflammatory factors, the MSC therapy can prevent the storm release of cytokines by the immune system and promote endogenous repair through their immunomodulatory, anti-inflammatory, and reparative properties [22,23].

Furthermore, the therapeutic potential of MSCs, largely mediated by paracrine signaling [24], is currently under investigation for several degenerative, autoimmune, and inflammatory disorders, as well as the exact mechanisms underlying their effect [21]. Nevertheless, it is very likely that either a direct cell-cell contact and/or the secretion of soluble factors, including bioactive lipids, and/or extracellular vesicles (EVs) are needed [24,25,26]. 

Generally, MSCs can modulate both innate and adaptive immune responses in vitro and in vivo due to their ability to inhibit T-cell proliferation and dendritic cell maturation, recruit regulatory T-cells, and modulate B-cell functions [21,27,28]. Expanded for the first time from human bone marrow (BM), MSCs can also be collected and cultured from several sources including adipose tissue, skeletal muscle, or umbilical cord blood and expanded ex vivo for clinical use [29,30]. Compared to BMs, adipose-derived stem cells (ASCs) have an easier and faster growth in culture, age with a lower rate, maintain the mesenchymal pluripotency and stem cell phenotype even after a high number of passages in culture, and show a great proliferative rate with a consequent relatively high yield (about 2500 fold higher than BM) [15,16,31,32]. Moreover, ASCs have shown a great potential of differentiation into several cellular lineages and a good stability throughout long-term cultures; they are characterized by immunomodulatory properties making them immunosuppressive [30]. Moreover, their secretome presents a mix of cytokines, extracellular matrix molecules and proteases, lipid mediators, hormones, and growth factors that are also involved in the angiogenesis process with a great utility and applicability in wound healing and tissue regeneration [15,33,34,35]. In addition to BMSCs and ASCs, skeletal muscle-derived stem cells (MDSCs) have been used in clinical trials for the regeneration and repair of injured tissues, because of their high proliferation rate and their ability to secrete trophic factors promoting endogenous tissues repair [36]; moreover, MDSCs harvesting consists in micro-biopsies obtained as small skin punctures under local anesthesia [37,38]. Although they exhibit slow-growing adherent behavior after isolation, MDSCs are characterized by a long-term self-renewal, and an easy differentiation into osteoblasts, adipocytes, and chondrocytes in vitro [37].

### 1.3. Functional Role of Endogenous Bioactive Lipids in Inflammation

Endogenous bioactive lipids cover a pivotal role in very important biological phenomena, such as inflammation, immune regulation, and maintenance of homeostasis [39,40]. Indeed, defects in their metabolism and unbalanced biosynthesis are involved in the pathogenesis and clinical course of chronic inflammation diseases [39,40]. Based on their biosynthesis, bioactive lipids can be grouped into different families (Table 1): polyunsaturated fatty acids (PUFA)-derived mediators (generally known as eicosanoids), endocannabinoids (eCBs), and lysophospholipids (LPLs) [41]. Bioactive lipids derived from PUFA can be further divided into two subgroups: one is represented by ω6 arachidonic acid (AA, 20:4 ω6)-derived lipid mediators, including prostaglandins (PGs), leukotrienes (LTs), thromboxanes (TXs), and lipoxins (LXs); the other includes ω3-PUFA-derived lipid mediators, such as the eicosapentaenoic acid (EPA, 20:5 ω3) and the docosahexaenoic acid (DHA, 22:6 ω3), i.e., E-series and D-series resolvins (Rvs), protectins (PDs), and maresins (MaRs), collectively termed “specialized pro-resolving mediators” (SPMs). Except for LXs, ω6-PUFA-derived lipids are pro-inflammatory, in contrast with ω3-PUFA-derived lipids, which act as anti-inflammatory. In detail, SPMs stimulate key cellular events, by acting as agonists, stopping further neutrophil influx and the activation of non-phlogistic responses by macrophages and, therefore, leading to the resolution of the inflammation. ECBs and eCB-like compounds originate from ω6- and ω3-PUFA metabolism, but also from saturated and monounsaturated fatty acids (SFA and MUFA), such as palmitic (16:0), stearic (18:0), or oleic acids (18:1 n9). Nowadays, pro- and anti-inflammatory properties exerted by eCBs and eCB-like compounds are issues of intense research [42,43]. Finally, membrane-derived bioactive lipids derived from LPLs can be divided into lysoglycerophospholipids (LGPLs) and lysosphingophospholipids (LSLs), based on the presence of glycerol or sphingosine (S) as backbone of their structures. LPLs exert pleiotropic effects such as inflammation, vesicular trafficking, endocytosis, apoptosis, cell migration, and cell-stress responses [44]. In this review, we will outline the biological activities and metabolisms of the major bioactive lipids identified as essential regulators in the complex scenario of inflammation and as players in the immunoregulation exerted by MSCs.

## 2. Lipids as Signaling Mediators in Inflammation 

### 2.1. Eicosanoids

**Figure 1:**
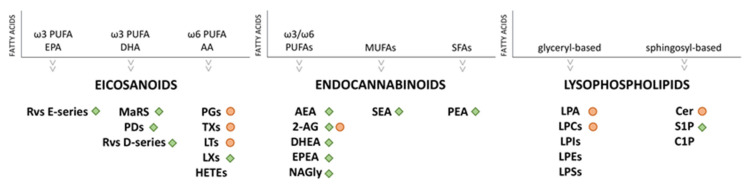
Lipids involved in inflammation. The green squares indicate lipids with anti-inflammatory properties and the orange dots indicate lipids with pro-inflammatory properties. Abbreviations. PUFAs: polyunsaturated fatty acids; MUFAs: monounsaturated fatty acids; SFAs: saturated fatty acids; EPA: eicosapentaenoic acid; DHA: docosahexaenoic acid; AA: arachidonic acid; Rvs E-series: resolvins E-series; Rvs D-series: resolvins D-series; MaRS: maresines; PDs: protectins; PGs: prostaglandins; TXs: thromboxanes; LTs: leukotrienes; LXs: Lipoxins; HETEs: hydroxyeicosatetraenoids; AEA: anandamide; 2-AG: 2-arachidonoilglycerol; DHEA: N-docosahexaenoylethanolamine; EPEA: N-eicosapentaenoylethanolamine; NAGly: N-arachidonoylglycine; SEA: stearoylethanolamide; PEA: N-palmitoylethanolamide; LPCs: lysophosphatidylcholines; LPA: lysophoshatidic acid; LPIs: lysophosphatidylinositols; LPEs: lysophosphatidylethanolamines; LPSs: lysophosphatidylserines; Cer: ceramide; S1P: sphingosine 1-phosphate; C1P: ceramide-1-phosphate.

The group of eicosanoids represents the widest family of bioactive lipids and includes several molecules characterized by the long carbon chain ω6 AA or ω3 EPA and DHA as common precursors. ω6 AA, released from membrane phospholipids firstly via phospholipase A2 and secondarily by phospholipase C, is the substrate for three different enzymes leading to the generation of pleiotropic and heterogenous compounds: (1) cyclooxygenases 1 and 2 (COX-1/2) drive the synthesis of PGs (PGD_2_, PGE_2_, PGI_2_, and PGF_2α_), prostacyclins, and TXs [45,46], also known as prostanoids; (2) 5-, 12- and 15-lypooxygenases (5/12/15-LOX) synthetize LTs [47,48], lipoxins (LXs) [49] and hydroxyeicosatetraenoids (HETEs) [50]; (3) P450 epoxygenase generates also HETEs, and epoxyeicosatrienoids (ETEs) [50]. ω3 PUFAs-derived bioactive products are Rvs, PDs, and MaRs. Rvs derive from either EPA or DHA and can be further divided into E-series or D-series, respectively. DHA acts also as a precursor for the biosynthesis of PDs and MaRs (Figure 1). The ω6 eicosanoids play an essential active role in the inflammatory response, such as leukocyte chemotaxis and activation, fever, pain [40], and are usually associated to acute inflammatory processes and chronic inflammation. Indeed, PGs seem to promote inflammation through several mechanisms such as increasing the release of the pro-inflammatory cytokines [51,52,53], enhancing the expression of pro-inflammatory genes, promoting innate immunity response [54], recruiting leukocytes and activating two distinct T helper subsets, TH1 and TH17 [55,56]. LTs generally recruit neutrophils, macrophages, eosinophils, and TH17 lymphocytes, and are responsible for the induction of edema. Vasoconstriction and vasodilatation are promoted instead by TXs and prostacyclins, respectively [57]. On the other hand, the ω3 family seems to have a beneficial impact on inflammation, by acting via different mechanisms, for example by working as substrate competitors able to inhibit the conversion of AA into pro-inflammatory eicosanoids or serving as an alternative substrate to produce less potent LTs, PGs, and TGs. In animal models, Rvs and PDs shorten the resolution of inflammation for certain diseases [41,58,59] and can also increase animal survival [60,61]. Two of the major Rvs, RvD1 and RvD2, have shown in vivo anti-inflammatory and pro-resolution properties, by blocking the neutrophil infiltration in many disorders, such as obesity and pathologies affecting the vascular [62], renal and dermal systems, and also in processes as wound healing, fibrosis, and pain [60]. Moreover, ω3 PUFA-derived mediators that have been found within the inflammatory exudate (RvE1 and PD1) show great anti-inflammatory and pro-resolving actions both in vitro and in vivo [51,63]. However, the resolution of inflammation is also mediated by other metabolites of AA [64]. Indeed, PGJ (15-deoxy-delta-13,14-PGJ_2_), the bioconversion product of PGD_2_, increases during the resolution phase and acts as a brake on inflammation by inducing apoptosis of inflammatory cells [65]. The concentration of the lipoxygenase product LXA_4_ (lipoxin A_4_) is also increased during the resolution phase and acts as a stop signal for the acute response [66]. Finally, AA-derived EETs present anti-inflammatory properties through the suppression of nuclear factor kappa-light-chain-enhancer of activated B cells (NF-kB) activation and govern vasorelaxation and fibrinolysis [67].

### 2.2. Endocannabinoids (eCBs) and Endocannabinoid-Like Compounds

eCBs are endogenous lipid compounds that can bind G-protein coupled cannabinoid receptors (CB1 and CB2) in the same way as tetrahydrocannabinol (THC), the major psychoactive component of *Cannabis sativa*. The plant *Cannabis sativa* and its preparations, marijuana and hashish, are being used for many years for recreational and medical purposes [68] because of the pleasurable effects triggered by THC, modulated by the other major, non-psychoactive phytocannabinoid, called cannabidiol (CBD). Both components possess other important medical properties, such as anti-inflammatory, analgesic, anti-emetic (THC), and anxiolytic (CBD). [69]. Thanks to the studies performed on cannabis plants and their peculiar chemical components, researchers were able to discover one of the most intriguing and pleiotropic endogenous signaling systems, the endocannabinoid system (eCBS). eCBs, CB receptors, and the biochemical entities that produce and degrade these lipids, are involved in most aspects of the mammalian physiology and pathology [70]. The compound arachidonoylethanolamide (AEA) [71], the first isolated ethanolamide of AA, represents a partial agonist of CB receptors, while 2- arachidonoylglicerol (2-AG) (another derivative of AA) [72,73], is a full agonist (Figure 1). Both compounds AEA and 2-AG belong to the group of PUFA AA-related lipid mediators, and as CB receptor ligands, they stimulate a variety of bioactivities, including analgesia, catalepsy, hypolocomotion, and hypothermia [68]. Moreover, AEA exhibits anti-inflammatory properties [74], whereas 2-AG shows both pro- and anti-inflammatory characteristics [75,76]. Thus, dysfunctions leading to changes in concentration levels, metabolism, and receptors of eCBs could be related to alterations in homeostasis and to the progression of chronic inflammatory status [77]. Moreover, two metabolically active ω3 fatty acid ethanolamides, N-eicosapentaenoylethanolamine (EPEA) and N-docosahexaenoylethanolamine (DHEA) [78,79], have been proposed as additional CB receptor agonists [80]. These ω3 eCBs were found to possess anti-inflammatory properties in macrophages [81] and adipocytes [82]. In addition to CB1 and CB2 receptors, pharmacological studies suggest the presence also of different receptors that can mediate the cannabinoids effects. Indeed, besides AEA, other ethanolamides coming from various long-chain fatty acids were discovered, and collectively known as N-acylethanolamines (NAEs). Ethanolamides of SFA and MUFA such as palmitic, stearic, and oleic acids, which are more abundant than AEA in mammals, show no activity for CB receptors, but act on other receptors, like the nuclear receptor peroxisome proliferator-activated receptor-α (PPARα), leading to the trigger of biological events including anti-inflammation and appetite suppression [83,84]. In detail, the PPARα-mediated actions of N-palmitoylethanolamide (PEA) include anti-inflammatory, analgesic, anti-epileptic, and neuroprotective properties [85,86]. Moreover, PEA could also activate the orphan G protein-coupled receptor GPCR55 [87], one of the discussed candidates as CB3 receptor, even though this agonist activity has not been fully elucidated yet. Another saturated NAE, N-stearoylethanolamide (SEA), was reported to act as an anti-inflammatory/immunomodulatory agent and cell growth controller, through still unknown targets [88,89,90]. Finally, a variety of eCB-related compounds, containing fatty acid chains conjugated with different polar heads, have been discovered as a result of advancements of the analytical techniques [91,92]. Within the novel group of lipids generally referred as lipoamino acids, N-arachidonoylglycine (NAGly), the most important member, possesses anti-inflammatory effects by targeting the G-protein coupled receptor GPCR18 [93,94], vasorelaxant properties [95] and seems to be involved in cell migration [96], and inhibition of the fatty acid amide hydrolase (FAAH) [97], the AEA inactivating enzyme. Moreover, NAgly might have either a physiological role in the resolution of acute inflammatory response and become a potential therapeutic candidate for the resolution of chronic inflammation, by increasing the production of PGJ and LXA_4_, reducing the migration of inflammatory cells into areas of acute inflammation and inducing the death of inflammatory cells [93].

### 2.3. Lysophospholipids (LPLs)

LPLs are bioactive signaling lipids consisting of *O*-acyl chain, generated from phospholipase-mediated hydrolyzation of membrane glycerophospholipids (GPLs) and sphingolipids (SLs). Consequently, LPLs are classified into two main categories: glyceryl-based LPLs (including LPA) and sphingosyl-based (including S1P) with a glycerol or a sphingosine backbone, respectively [98,99] (Figure 1). Several LPLs compounds are asymmetrically distributed in the plasma membrane and are characterized by a polar head group (ethanolamine, choline, inositol, serine) and a hydrophobic tail of carbon chain. LPLs act as signaling mediators by binding seven-transmembrane domain G-protein coupled receptors (GPCRs). The two major bioactive LPLs are the well-characterized lysophosphatidic acid (LPA) and sphingosine-1-phosphate (S1P) and they play important roles in various physio-pathological processes, including inflammation. LPA, a byproduct of lysophosphaditylcholine (LPC) and lysophosphatidilinositol (LPI), is a signaling mediator involved in cell renewal, immune response, and inflammatory cascade [100,101]. LPA can be synthetized both intracellularly and extracellularly by different enzymes and via different pathways, such as autotaxin/ectonucleotide pyrophosphatase phosphodiesterase 2 (ENNP2) and/or phospholipases A1 and A2, whereas its degradation is mediated by lipid phosphate phosphatases 1–3 [102]. Currently, six LPA receptors (LPA 1–6) are known [102]. Recently, LPA is reported to be rapidly formed during the resolution phase of the inflammation and, successively, to be recruited via the common pro-resolving formyl peptide receptor 2 (FPR2, also known as ALX), which is expressed on T cells and their subsets [103]. On the other hand, SLs, such as ceramides and sphingosines, participate in different stages of inflammation as well, by controlling intracellular trafficking and signaling, cell proliferation, adhesion, vascularization, survival, and apoptosis [104,105]. In particular, the phosphate forms of sphingolipids, ceramide-1-phosphate (C1P) and S1P [106], are notably associated to inflammatory responses. S1P is synthesized by the intracellular phosphorylation of sphingosine via sphingosine kinases 1 and 2 (SK1 and SK2) and degraded by S1P lyase or ceramide synthases. It is involved in the resolution phase (together with C1P) since apoptotic cells present at the inflammation sites attract pro-resolving macrophages via S1P receptor 1 [107] and, additionally, it can act either on COX-2 or NF-kB, whereas C1P acts on phospholipase A2 [102].

## 3. Bioactive Lipids in MSCs

### 3.1. Lipid Metabolism in MSCs Maintenance and Differentiation

**Figure 2:**
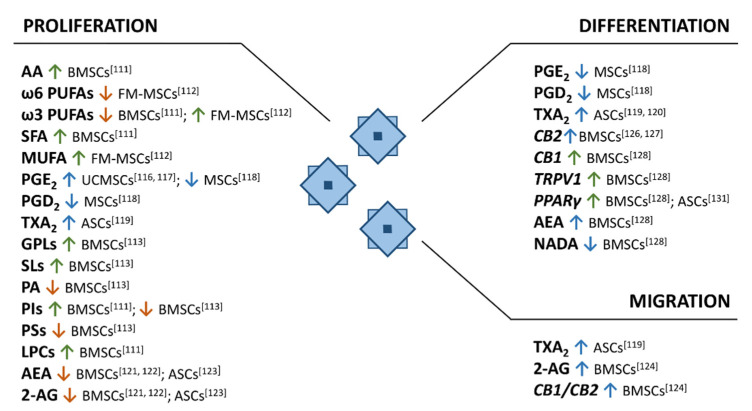
Lipids and their receptors involved in MSCs proliferation, differentiation and migration. The green and orange arrows indicate an increment and a decrease of lipids or their receptors, respectively; whereas blue arrows indicate their action on MSCs. Abbreviations. AA: arachidonic acid; PUFAs: polyunsaturated fatty acids; SFAs: saturated fatty acids; MUFAs: monounsaturated fatty acids; PGE_2_: prostaglandin E_2_; PGD_2_: prostaglandin D_2_; TXA_2_: thromboxane A_2_; GPLs: glycerophospholipids; SLs: sphingolipids; PA: phoshatidic acid; PIs: phosphatidylinositols; PSs: phosphatidylserines; LPCs: lysophosphatidylcholines; AEA: anandamide; 2-AG: 2-arachidonoilglycerol; CB2: cannabinoid receptor 2; CB1: cannabinoid receptor 1; TRPV1: transient receptor potential vanilloid type 1; PPARγ: peroxisome proliferator-activated receptor-γ; NADA: N-arachidonoydopamine; BMSCs: bone marrow-derived stem cells; FM-MSCs: fetal membrane-derived stem cells; UCMSCs: umbilical cord blood-derived stem cells; ASCs: adipose-derived stem cells.

Lipid metabolism plays a pivotal role in stem cells physiopathology [108,109,110]. However, at the moment the number of studies about the lipidome of MSCs is limited, and mainly focused on variations in lipid composition during stem cell proliferation and differentiation [111,112,113,114,115,116,117,118,119,120,121,122,123,124,125,126,127,128,129,130] (Figure 2).

Recently, profiles of glycerophopholipids (GPLs) present in human BMSCs were assessed from young and old donors and across passages during in vitro culture [111,112,113]. In particular, since the clinical use of MSCs demands sequential ex vivo expansion, the determination of GPL profiles through the different steps of the in vitro culture represents a crucial and relevant advancement. In general, long-term culturing could contribute to the decrease of the proliferation and the differentiation potential, shorten the telomers, and accumulate ω6 PUFAs with signaling roles, consequently promoting inflammation [114,115]. It is well established that membrane GPLs provide precursors for signaling lipids that modulate cellular functions, and small changes in their compositions can lead to significant biological consequences. Kilpinen et al. studied the effect of the donor’s age and cell doublings on the profile of GPLs of human BMSCs, demonstrating that an extensive expansion modulates membrane GPLs, by increasing total phosphatidylinositol (PI) and lysophospatidylcholine (LPC). Specifically, the effect was more pronounced when BMSCs were isolated from young donors. Moreover, changes in membrane FAs profile during expansion and senescence of BMSCs was highlighted: the ω6 AA content increased, while ω3 PUFAs (especially DHA) decreased during long-term cultivation, leading to an impairment of the immunological functionality [111]. In addition, in the later steps of the process, an increment of the fraction of individual SFA was noticed [111]. A significant modification of membrane FAs composition of MSCs derived from human fetal membranes (FM-MSCs), occurring during in vitro culture, was assessed by Chatgilialoglu et al. [112]. In detail, fresh uncultured FM-MSCs showed variability in their membrane FAs composition, likely due to the genetic diversity and different lifestyle of the donors. This study also reveals that cultured cells have lower proportions of PUFAs than freshly isolated cells showing a great drop in ω6 FAs, counterbalanced by a marked increase in MUFA and ω3 FAs. These data are in contrast with Kilpinen et al. [111]. More recently, a lipidomics profiling analysis during BMSCs culturing passages by Lu et al. investigates the metabolic alteration of various lipid species in the senescence process [113]. They applied an untargeted lipidomics approach based on liquid chromatography coupled to mass spectrometry (HPLC-MS), which allowed the reduction of the complexity of the matrix and the enhancement of the sensitivity, factors that represent an improvement relative to the previously described shotgun-based methods. The majority of GPLs, as well as SLs, were found to significantly increase across the culturing passages, whereas the PA, PIs, and phosphatidylserines (PSs) levels were lower in aged cells. These findings were largely coherent with previous described studies, except for PI species, which were found to be increased during all the passages [111]. Nevertheless, the reduced amount of PIs is inconsistent with the relative transcriptomics analysis, which showed an increase in the enzymes expression with consequential conversion of PA into PIs suggesting an enhanced PIs biosynthesis activity. Moreover, research on the functional FAs has largely supported regulatory roles for PGs in MSCs proliferation. In particular, PGE_2_ increases human umbilical cord blood-derived MSCs (UCMSCs) proliferation through β-catenin-mediated c-Myc and vascular endothelial growth factor expression via exchange protein directly activated by cAMP (Epac1)/Ras-related protein 1 (Rap1)/Akt and PKA cooperation [116], and through interaction of profilin-1 (Pfn-1) and filamentous-actin (F-actin) via EP2 receptor-dependent β-arrestin-1/JNK signaling pathways [117]. On the contrary, the investigation of PGE_2_ and prostaglandin D2 (PGD_2_) effects on MSCs proliferation and osteogenic differentiation suggests that both their receptors are highly expressed in these cells and both prostaglandins seem to have a negative impact [118]. In detail, PGE_2_ firstly enhances the MSCs growth-rate, while longer stimulation leads to a growth-inhibitory effect. Contrarily, PGD_2_ inhibits MSCs growth regardless of the duration of the exposure. Moreover, their inhibitory effect on calcium deposition also suggests a negative impact on MSCs osteogenic differentiation [118]. Moreover, TXs class has been investigated for its effect on MSCs proliferation, suggesting the role of TXA_2_ as potent modulator of ASCs migration and proliferation through ERK and p38 MAPK signaling mechanisms [119]. In addition, TXA_2_ appears to induce ASCs differentiation into smooth-muscle-like cells [119,120]. Concerning eCBS, Rossi et al. [121] described a gradual decrease during subculture in AEA and 2-AG levels secreted by human BMSCs starting from passage 1 (AEA: 5 pmol/mg protein and 2-AG: 11 pmol/mg protein 2-AG) and this finding was also confirmed by Kose et al. [122]. ASCs secrete AEA and 2-AG at 3.5 and 7.3 pmol/mg protein, respectively, at early passages [123]. In addition, 2-AG and CB1/CB2 stimulation recruits BMSCs, most probably via an indirect activation of CB2 receptors [124].

During MSCs differentiation, eCBS variation was also highlighted and the expression of CB1 and CB2 is considerably lower in undifferentiated cells and it increases during osteogenic [125,126] and adipogenic commitment [125]. Furthermore, the activation of CB2 signaling plays an important role in promoting the osteogenic differentiation of BMSCs in vitro, with an increase of alkaline phosphatase activity (ALP), an induction of the expression of specific osteogenic genes including Runx2, Osterix, IBSP, SPP1, OCN, COL1a1, and an enhanced deposition of calcium in the extracellular matrix [126]. This result indicates a key role of CB2 receptor in BMSCs differentiation towards osteoblasts, suggesting also that MSCs might produce endogenous cannabinoids able to reinforce their osteogenic differentiation as well. Moreover, the knockdown of CB2 receptor in BMSCs by small interference RNA (siRNA) inhibits ALP activity and mineralization [126]. Most recently the osteogenic differentiation induced by CB2 signaling activation has been shown to involve autophagy induction and sequestosome 1/p62-mediated Nuclear Factor Erythroid 2Related Factor 2 deactivation [127]. Whereas, the implication of eCBs in BMSCs physiology related to their adipocyte differentiation was validated looking at the increased expression of CB1, transient receptor potential vanilloid type 1 (TRPV1) and PPARγ during adipogenesis [128]. Moreover, the effects of AEA, N-arachidonoydopamine (NADA), and 2-AG were evaluated suggesting a promotion of adipocyte differentiation by AEA and an inhibition by NADA. No changes were observed with 2-AG at non-cytotoxic concentrations. Furthermore, CB1 may stimulate protein expression, such as adiponectin during adipogenesis [125,129], since it is enriched in mature adipocytes compared to other cell types [129]. Moreover, based on the effect of AEA, CB1 expression seems to be correlated to the increment of FAAH and COX-2 during adipogenic differentiation [130].

In addition, Pagano et al. found out that ASCs exposed to the synthetic cannabinoid WIN55,212–2 increase the glucose uptake, the calcium influx, and the expression of the adipogenesis regulator PPAR-γ; contrarily, these effects are inhibited by the specific CB1-antagonist Rimonabant [131]. Finally, Silva et al. has analyzed the lipidome of rabbit ASCs and MDSCs and their adipogenic and osteogenic differentiation identifying 1687 lipid species [132]. These animal MSCs have shown different lipid profiles as well as changes in lipid composition after adipogenic and osteogenic differentiation. Moreover, the N-acyl-phosphatidylethanolamine (PE) and phosphatidylcholine (PC) expression levels suggest lipid similarities in cells differentiated from different stem cell sources [132]. In conclusion, PUFAs and their bioactive derivatives affect both the proliferation and differentiation of several MSCs and consequently modulate their immunological interaction with other cells. In this perspective, lipid profiling can represent a valuable tool also in the screening of MSC populations prior to their use in both experimental and clinical settings. Indeed, the possibility of evaluating selected lipid classes or MSC entire lipidome can rapidly provide a screenshot of their differentiative status and growth rate, thus, allowing to harness MSC potential at its best for the diverse applications.

### 3.2. Pro and Anti-Inflammatory Properties of MSC-Derived Lipids

MSCs present anti-inflammatory properties and are being used with great success as treatment for inflammatory and autoimmune diseases. They have been shown to migrate towards injured tissues affected by inflammatory events, led by several growth factors, cytokines, and chemokines [133]. Being physiologically recruited at the damaged site, MSCs are often submitted to a strong, pro-inflammatory environment. It is well known that the PGE_2_ secretion is increased upon incubation with the tumor necrosis factor alpha (TNF-α) and the interferon gamma (IFN-γ) [28]. To better understand the involvement of the lipidome in the MSCs anti-inflammatory properties and underlying its mechanisms of action, Campos et al. [134] have performed a wide range lipidomic analysis of MSCs under pro-inflammatory conditions induced by the presence of 10 ng/mL TNF-α and 500 U/mL IFN-γ. This study has evidenced a change in MSCs PL profile under the pro-inflammatory stimulus: indeed, higher levels of molecular PC species with longer FA acyl chains and lower levels of molecular PC species with shorter FA acyl chains were assessed. Moreover, the expressions of the specific PE(40:6), PS(36:1), LPC(18:0), and SM(34:0) were enhanced, while PE(O-38:6) and PS(40:4) expressions decreased simultaneously. The increase of LPC (18:0) has already been correlated with anti-inflammatory properties by others [135,136]. These differences were identified only in specific GPL subspecies, suggesting that each GPL subspecies could play a role in MSCs immunological functions. Moreover, the characteristics of the lipidome of the untreated MSCs described by Campos et al. were consistent with previous results [111], with the exception of the presence of sphingomyelins [134], which have not been previously identified. As formerly described, some derivatives of SLs, such as S1P, are bioactive and mediate essential cell functions [137].

Concerning the MSCs lipid secretion, PGE_2_ was widely investigated given its key role in the immunosuppressive activity of MSCs [28]. Masoodi and colleagues [138] have analyzed the release of PGs by human heart-derived MSCs by HPLC-MS/MS, finding the presence of PGE_1_, PGE_2_, PGE_3_, 6-keto PGF_1α_, PGF_2α_, and PGJ_2_ in the conditioned medium. Although PGE_2_ has been linked to the immunosuppressive effects of MSCs since their inhibitors production attenuate MSC-mediated immunomodulation [28], PGs are best known for their ability to mediate vasodilatation that allows immune cells to invade inflamed tissue. Indeed, recent evidence suggests also that PGE_2_ may have an immunostimulatory role by facilitating Th1 differentiation and expanding the Th17 T-cells population [55]. Since prostaglandins have a short half-life, they act as paracrine and autocrine factors in the local environment. MSCs themselves also express receptors for prostaglandins: EP1, EP2, EP4, FP, and IP. The effects triggered by the stimulation of these receptors on MSCs are still unknown. However, the profile of PGs highlighted in MSCs is superimposable with that of their receptors (prostaglandins type E and F, and prostacyclin). Thus, the dual and controversial immunomodulatory properties of MSCs can depend on the local environment, where IFN-γ and TNF-α play a pivotal role in promoting immunosuppressive function of MSCs [139,140].

In the presence of PGE_2_, also a higher expression of EP3, which is involved in the stimulation of angiogenesis, was obtained in MSCs suggesting a possible correlation with the early phases of inflammation [118].

Recent studies have evidenced the roles of LXs as regulators of the resolution phase of inflammation [61] and of Rvs as players in the immunoregulation of MSCs [141]. Fang et al. have demonstrated the MSCs ability of promoting the resolution of acute lung injuries in mice through the secretion of lipoxin A_4_ (LXA_4_), the first identified anti-inflammatory and pro-resolving lipid mediator [142], signaling via the G protein coupled ALX/FPR2 receptors [141].

### 3.3. Effect of Exogenous Supplements of PUFAs on MSCs

The ω3 fatty acids EPA and DHA, which are found mainly in marine oils, have long been thought to have anti-inflammatory properties, whereby they compete with AA, by reducing pro-inflammatory eicosanoids [143]. The molecular mechanism through which this occurs is still unclear, and there are no evidences about beneficial effects of ω3 EPA and DHA for human health as well as their role as potential treatments for human diseases. In most mammalian cell types, different exogenous supplements of PUFAs are incorporated into plasma membrane GPL and then metabolized by phospholipases in order to produce various lipid mediators. Thus, the biochemical homeostasis of lipid profile in mammalian membranes must be perturbed not only by physio-pathological inputs, but also by external lipid uptake (i.e., dietary fats). A recent study performed on human BMSCs has demonstrated the increase of the secretion of the pro-inflammatory PGE_2_ after AA supplements intake. However, this possible harmful effect can be attenuated by the chain elongation on the less active precursor, ω6 22:4. The ω3 PUFAs precursor, the alfa-linolenic acid (18:3), shows a slight reduction of its GPL AA content, while the EPA (20:5) and DHA (22:6) acid supplements efficiently displace the AA, creating several pools of GPL species substrates that allow attenuation of inflammatory signaling [144].

### 3.4. MSCs as an Alternative Treatment of Inflammatory Diseases: The Example of Osteoarthritis

Osteoarthritis (OA) is a heterogeneous chronic joint disease characterized by the processes of degradation, repair, and inflammation that occur in the connective tissue, the vulnerable layer of joints, synovium, and subchondral bone [145]. From a molecular point of view, the catabolic and anabolic activities are unbalanced, and the major injury response occurs at the joint cartilage level. Recently, findings regarding the involvement of lipids in OA development and progression indicate a possible involvement of ω3 PUFAs and their anti-inflammatory SPMs derivatives [146]. The most studied bioactive lipids, PGs and LTs, have been detected in plasma and synovial fluid of OA patients showing pro-inflammatory and catabolic effects on fibroblasts, osteoblasts and cartilage [147]. Moreover, the PGE_2_ and AA-derived oxylipin 15-HETE levels were related to knee OA [148], suggesting a possible role in the disease progression. Because of the similarities between OA course and chronic wound accompanied by cell death, inflammation, and pain [149] and since ω3 PUFAs/SPMs have been shown to target all these processes, it is conceivable that these lipids could be effective therapeutic agents for OA. In the context of this disease, few studies have investigated the FAs presence in OA affecting patients and their relationship to clinical symptoms. These studies indicated that increases of ω3 FAs levels could be associated with a reduced cartilage loss while the increase of the increase of ω6 FAs levels with enhanced synovitis [150]. All studies performed with ω3 PUFAs suggest that the beneficial effects consist primarily in an improvement in symptoms and pain, whereas little effects are observed on structural progression of the OA disease. However, previous studies have reported that ω3 PUFAs can counteract the pro-inflammatory and catabolic actions of interleukin-1a (IL-1a) on cartilage in vitro [151]. These results were consistent with a more recent study in which the authors have shown the involvement of DHA in the downregulation of MMP-13 through a P38 mitogen activated protein kinases (p38-MAPK)-mediated mechanism [152] both in vitro and in vivo in a rat model of OA. Apart from direct effects of ω3 PUFAs on OA, it is conceivable that ω3-derived oxylipins could be generated in vitro (i.e., by chondrocytes) and these could mediate the observed effects. Another study confirmed the presence of pro-inflammatory lipid mediators, such as PGE_2_, in OA synovial fluid, as well as oxylipins derived from ω3 and ω6 PUFA such as 15-HETE (derived from AA), 17-HDHA (derived from DHA), and 18-HEPE (derived from EPA). When the pro-inflammatory response occurs in the cartilage, some types of prostanoid enzymes, such as COX, will be produced and released in excessive amounts. COX activation will increase the production of MMP, inhibit the expression of PGE_2_ and collagen genes and will stimulate the apoptosis process. Studies conducted by Hardy et al. [153] and Shimpo et al. [154] have analyzed the role of PGE_2_ in chondrocytes. The pro-inflammatory cytokine IL-1β stimulates the production of PGE_2_ in large quantities, and this could induce the degradation process of OA. At the molecular level, IL-1β will increase the expression of the COX-2 gene and the microsomal prostaglandin E synthase-1 at mRNA and protein levels. Therefore, an increase in PGE_2_ production is related to mPGES-1 and COX-2 derivatives from osteoarthritis chondrocytes stimulated by IL-1β. Another recent study has shown the beneficial effects of resolvin D1 on OA chondrocytes. RvD1 belongs to the family of D-series Rvs, which includes RvD2–RvD6 and share the common precursor 17-HDHA. In one study, RvD1 was found to inhibit the IL-1β-mediated upregulation of COX-2, PGE_2_, MMP13, and nitric oxide and to prevent chemically induced apoptosis in human osteoarthritis chondrocytes [155]. These effects are mediated by the downregulation of the nuclear factor NF-kB, p38-MAPK, and c-Jun N-terminal kinases activation, as well as inactivation of caspase9 and upregulation of Bcl-2 and Akt. Despite the high concentrations of RvD1 used in this study (mM range), these data indicate for the first time the potency of an SPM to counteract deleterious processes in OA chondrocytes. MSCs have been demonstrated to be effective in the treatments of different tissue injuries and, in particular, they have been considered as a promising alternative cell source for cartilage repair [156]. However, recent studies have suggested that the beneficial effects of MSCs on injured tissues could be attributed to the activation of a protective mechanism and the stimulation of endogenous regeneration rather than to their differentiation potential [157]. MSC-secreted bioactive molecules and/or EVs may act as paracrine or endocrine mediators that directly activate target cells or neighboring cells to secrete functionally active agents. Indeed, we recently demonstrated the therapeutic potential of ASCs secretome and EVs both in vitro on TNFα-stimulated articular chondrocytes [158,159], and in vivo in a mouse model of OA [160], providing evidences of MSC mediated anti-inflammatory and immunomodulatory action. Consistently, the influence of MSCs towards PGE_2_ gene expression was studied in the pathogenesis of OA. One study showed that MSCs could significantly (*p* < 0.05) reduce PGE_2_ expression in OA synoviocytes after 24 and 48 h co-culture compared to control cells [161]. Moreover, several researches disclosed that MSC-derived EVs stimulate tissue regeneration [162], and EVs have generally important functions in cell communication and regulation. EVs are home to the inflammatory site and transfer proteins/peptides, mRNA, microRNA, lipids, or organelles with reparative and anti-inflammatory properties [161,163]. Lipids are essential components of the EVs membranes, and it is well known that specific lipids are enriched in EVs compared to their parent cells. For example, it has been shown a 2–3 times enrichment from cells to EVs for cholesterol, GPLs, and PSs [164,165]. On the contrarily, EVs generally contained less PCs than their parent cells. At the moment, the physiological importance of the asymmetric lipids distribution between EVs and parent cells is still largely unknown. Compared to the original BMSCs, Xiang et al. found out that MSC-EVs were highly enriched in the cell proliferation and migration mediator S1P by the involvement of sphingosine kinase 1 (SK1) [166]. In detail, human chondrocytes were co-cultured with MSC-EVs showing enhanced proliferation and decreased apoptosis induced by IL-1β, known as one of the main inflammatory mediators for arthritis. The highlighted MSC-EVs therapeutic effect occurs in part through the S1P/S1P receptor 1 (S1PR1) signaling pathway activation. So, also this study suggests the implication of lipids and their related pathways (i.e., S1P/S1PR1) into the clinical application of MSC-EVs to the treatment of articular cartilage defect. Future lipidomic research, aimed at characterizing the lipid mediators of the crosstalk among MSCs and other articular cell types (e.g., chondrocytes, synoviocytes, or osteoblasts), would likely uncover additional inflammatory pathways associated with OA, with interesting repercussions in the clinical management of this pathology.

## 4. Conclusions

In the last few years, lipidomics has gathered the interest of the scientific community because of the recently confirmed role of lipids in several biochemical aspects as first actors. In detail, lipids are recognized as key players in cells membrane and signaling processes, such as inflammation and immunomodulation. Furthermore, cell lipidome changes according to different cell phases and microenvironment features. Therefore, by analyzing differences in profiles of specific lipid species, it is possible to obtain insights regarding lipids interference in cell signaling and other cellular mechanisms. Lipidomics has proved being successful in identifying viable and functional cell cultures, which could guarantee efficient and safe MSCs application. Despite the limited availability of data regarding MSC lipidomics, the pleiotropic biological actions of different lipid families indicate them as promising candidates for future therapeutic interventions.

## Figures and Tables

**Table 1:** Composition, functions, and classes or examples of the different categories of bioactive lipids.

Categories	Composition	Function	Classes or Examples
Polyunsaturated fatty acids	Carboxylic acid + hydrocarbon chain; synthesized by chain elongation of an acetyl-CoA with malonyl-CoA	Cell signaling; building blocks to complex lipids	AA, EPA, DHA
Endocannabinoids and related compounds	Ethanolamide or other head groups + FAs	Cell signaling	AEA, 2AG
Lysophospholipids	Polar head group + glycerol or sphingosine backbone	Membrane and lipoprotein composition, cell signaling	LGPLs, LSLs

## Data Availability

No new data were created or analyzed in this study. Data sharing is not applicable to this article.

## Abbreviations

2-AG 2-arachidonoilglycerol; AA arachidonic acid; AEA anandamide; ALP alkaline phosphatase activity; ASCs adipose-derived stem cells; BM bone marrow; BMSCs bone marrow-derived stem cells; C1P ceramide 1-phosphate; CB cannabinoid receptors; CBD cannabidiol; COL1a1 collagen type I α 1; COVID-19 coronavirus disease 2019; COX cyclooxygenases; DHA docosahexaenoic acid; DHEA N-docosahexaenoylethanolamine; eCB endocannabinoids; EETs epoxyeicosatrienoic acids; EPA eicosapentaenoic acid; EPEA N-eicosapentaenoylethanolamine; ETEs epoxyeicosatrienoids; EVs extracellular vesicles; FAs fatty acids; FM-MSC fetal membrane-derived stem cells; GC gas chromatography; GPCR G protein-coupled receptor; GPLs glycerophospholipids; GPLs glycerophospholipids; HETEs hydroxyeicosatetraenoic acids; HPLC-MS liquid chromatography coupled to mass spectrometry; IBSP integrin-binding sialoprotein; IFN-γ interferon gamma; LC liquid chromatography; LGPLs lysoglycerophospholipids; LOX lipoxygenases; LPA lysophosphatidic acid; LPCs lysophosphatidylcholines; LPEs lysophosphatidylethanolamines; LPIs lysophosphatidylinositols; LPLs lysophospholipids; LPSs lysophosphatidylserines; LSLs lysosphingophospholipids; LTs leukotrienes; LXs lipoxins; MaRs maresins; MDSCs skeletal muscle-derived stem cells; MS mass spectrometry; MSCs mesenchymal stem cells; MUFAs monounsaturated fatty acids; NADA N-arachidonoyldopine; NAEs N-acylethanolamines; NAGly N-arachidonoylglycine; OA osteoarthritis; OCN osteocalcin; OSX osterix; PA phoshatidic acid; PDs protectins; PEA N-palmitoylethanolamide; PGs prostaglandins; PIs phosphatidylinositols; PPARα receptor peroxisome proliferator-activated receptor-α; PSs phosphatidylserines; PUFAs; polyunsaturated fatty acids; RUNX2 runt-related transcription factor 2; Rvs resolvins; S1P sphingosine 1-phosphate; SEA N-stearoylethanolamide; SFAs saturated fatty acids; SK sphingosine kinases; SLs sphingolipids; SPMs specialized pro-resolving mediators; SPP1 secreted phosphoprotein 1; THC delta9-tetrahydrocannabinol; TLC thin-layer chromatography; TNF-α tumor necrosis factor alpha; TRPV1 transient receptor potential vanilloid type 1; TXs thromboxanes; UCMSCs umbilical cord blood-derived MSCs; ω-3 PUFAs omega-3 polyunsaturated fatty acids; ω-6 PUFAs omega-3 polyunsaturated fatty acids.
